# Association Between the Location of Secondhand Smoke Exposure and Depressive Symptoms among South Korean Adolescents

**DOI:** 10.3390/ijerph17145116

**Published:** 2020-07-15

**Authors:** Bich Na Jang, Wonjeong Jeong, Soo Hyun Kang, Sung-In Jang

**Affiliations:** 1 Department of Public Health, Graduate School, Yonsei University, Seoul 03722, Korea; jbn2846@gmail.com (B.N.J.); wjjeong@yuhs.ac (W.J.); kshyun@yuhs.ac (S.H.K.); 2 Institute of Health Services Research, Yonsei University, Seoul 03722, Korea; 3 Department of Preventive Medicine, College of Medicine, Yonsei University, Seoul 03722, Korea

**Keywords:** secondhand smoke, depressive symptoms, adolescent health

## Abstract

The incidence of depression among adolescents has gradually increased, leading to adult psychological outcomes and suicide. Although the rate of secondhand smoke exposure (SHSE) has recently decreased, SHSE remains high in children. We aimed to determine the association between depressive symptoms in adolescents and the locations of SHSE using an extensive population survey. Using data from the 14th Korea Youth Risk Behavior Web-based Survey, we assessed self-reported data of depressive symptoms and SHSE among non-smokers. SHSE locations were classified into four groups: only at school, only at home, at both school and home, and other places. Multiple logistic regression analysis was performed to identify the associations between SHSE locations and depressive symptoms. The relationship between SHSE and depressive symptoms was the highest in the “SHSE at home and school” group (boys: odds ratio [OR] = 1.61, 95% confidence interval [CI] = 1.44–1.80; girls: OR = 1.72, 95% CI = 1.54–1.91), followed by the “school” (boys: OR = 1.53, 95% CI = 1.39–1.67; girls: OR = 1.36, 95% CI = 1.25–1.48) and “home” groups (boys: OR = 1.23, 95% CI = 1.12–1.35; girls: OR = 1.30, 95% CI = 1.20–1.40). These results emphasize the importance of stricter smoking regulations not only in public places, but also in households. Adolescents and their families should be educated on the dangers of smoking and the effects of SHSE on mental health.

## 1. Introduction

The number of adolescents with depressive symptoms has exceeded that of adults [1] and has gradually increased [1,2]. Adolescent depression is associated with adult psychological outcomes [3] and may lead to suicide [4,5], which is a serious public health issue [6]. The suicide rate in South Korea is the highest among those of the Organization Economic Co-operation and Development countries [7], trending upward from 2017 to 2018 [8]. Moreover, suicide is the leading cause of death in Korean adolescents [8].

Despite efforts to curb smoking rates at the government level, the adolescent smoking rate has remained consistent for three years. Smoking in adolescence is likely to continue into adulthood due to nicotine addiction and leads to adverse outcomes, such as cardiovascular disease, cancer, or respiratory disease [9,10]. In addition, adolescent smokers experience more stress than non-smoking adolescents [11]. A number of studies have shown a significant association between depression and smoking [12,13], emphasizing the importance of smoking cessation [14]. Furthermore, in non-smoking adolescents, exposure to secondhand smoke is associated with mental disorders, such as depression and anxiety [15,16,17], and this association was different according to gender [18].

It is widely established that secondhand smoke (SHS) causes adverse health effects similar to firsthand smoking [9]. SHS in adolescents leads to adverse outcomes in adulthood, such as cardiovascular dysfunction [19] and respiratory disease [20], and may increase mortality [21]. Although the rate of secondhand smoke exposure (SHSE) has decreased recently, SHSE remains especially high among children [22], who are more likely to be exposed to SHS in their home than in other locations, depending on the socioeconomic status of the household.

In the United States, those living below the poverty line were more vulnerable to SHSE [22], and similar results were noted in a study on Korean adolescents. Non-smoking adolescents who were reported their household income as low were more vulnerable to household secondhand smoke exposure (HSHSE) than those with high household incomes [18]. In Canada, school secondhand smoke exposure (SSHSE) remained high despite the implementation of smoke-free zone regulations [23].

Adolescents are easily affected by their surrounding environment [24], leading to either positive or negative health outcomes [25]. Unhealthy behaviors in adolescence, such as smoking and drinking, are modeled by peers or parents. However, the impact of the influence of peers or parents’ smoking status is different for adolescents [26,27]. As mentioned above, several studies have reported the risks of SHSE among adolescents, and the association between smoking and depression is well-established. A study reported an association between the location of SHSE and depression, but was limited by a small sample size [16]. Another study found a positive association between HSHSE and depression [28].

In the present study, we hypothesized that the location of exposure to secondhand smoke is related to depression, because adolescents are greatly affected by their surroundings. Therefore, our study’s objective is to determine the association between depressive symptoms and the locations of SHSE stratified by gender analyzed by multiple logistic regression. We further analyse the association related to smoking and socioeconomic status. Most of all, this study focused on SHSE locations, especially in the home and at school, among Korean adolescents.

## 2. Materials and Methods

### 2.1. Procedure

This study used data from the 14th Korea Youth Risk Behavior Web-based Survey (KYRBWS) which was carried out in 2018. The KYRBWS is a cross-sectional study conducted in collaboration with the Ministry of Education, the Ministry of Health and Welfare, and the Korea Centers for Disease Control and Prevention (KCDC). It is an anonymous self-reporting online survey for Korean middle and high school students (grades 7–12, mean age 15). The KYRBWS was approved by the KCDC Institutional Review Board (2014-06EXP-02-P-A) until the 10th survey. Since the 11th survey, the requirement for ethical approval for the KYRBWS was waived by the KCDC Institutional Review Board under the Bioethics & Safety Act, and the survey data is publicly available (Available at: http://www.cdc.go.kr/yhs/home.jsp). All participants provided informed consent to participate in the KYRBWS and were guaranteed anonymity.

Because this survey included about 60,000 participants per year, we used only one year, the 14th Survey. It is applied a cluster-sampling design with proportional allocation in each of 117 clusters to minimize sampling errors. It included 103 questions assessing demographic characteristics and health-risk behaviors in 15 areas, including smoking, alcohol consumption, weight control, physical activity, dietary behavior, injury prevention, violence, sexual behavior, mental health, oral health, allergic diseases, internet addiction, drug abuse, and health equity. The survey included 60,040 participants (boys: girls, 30,463:29,577) and the response rate was 95.6%.

Among the 60,040 participants, we excluded those who answered “don’t know” to the question, “indicate everyone in your family who currently smokes” as they might not experience secondhand smoke in their home. Subsequently, a total of 58,182 participants (boys: girls, 29,481:28,701) was selected for this study.

### 2.2. Variables

The outcome variable was depressive symptoms, which was assessed by the answer to the question, “in the last year, have you experienced sadness or hopelessness enough to stop your daily activities over 2 weeks?” Those who answered “yes” were classified as having a depressed mood and others were classified as no depressed mood. We used this question to identify depressive symptoms. A single-item question about depression has comparable performance features as a longer 20-item scale and is more feasible because of its brevity [29].

The primary independent variable was the location where adolescents were exposed to SHS. We used two different locations in the analysis: at home and at school. To determine SHSE, we assessed the following questions: “during the last 7 days, how many days did you stay with either your family or visitors while they smoked in your home?” and “during the last 7 days, how many days did you inhale other people’s smoke at school?” Those who answered “no experience in 7 days” to both questions were classified as those who had no experience with secondhand smoke in either location, and those who gave other answers were sorted into the SHSE group. Then, we separated the SHSE group into four subgroups by location: only at school (school), only at home (home), both at home and at school (both), or no exposure in either location (none).

Other independent variables that may act as potential confounding variables included sex, age, school, living with family or not, perceived economic status, smoking status, family/friends’ smoking status, alcohol consumption experience, physical activity, academic scores, and stress status. The present study included participants who currently or previously smoked, which was assessed by a combination of two questions: “during the last 30 days, how many days did you smoke at least 1 cigarette?” and “during the last 30 days, how many cigarettes did you smoke on average?” Those who answered “not applicable” for both questions were categorized as non-smokers, those who answered “did not smoke during the past 30 days” for the first question were categorized as ex-smokers, and the remaining participants were categorized as smokers. Since there was no question relating to exposure to nicotine, we considered it via the number of cigarettes smoked in a day, and we divided into two groups using the median value which was 5 cigarettes [30].

### 2.3. Statistical Analysis

Independent variables were compared using the Chi-square test to identify the association between SHSE the presence of a depressed mood. After adjusting for demographic, socioeconomic, and health-related variables, we used multiple logistic regression analysis to evaluate the association between SHSE location and depressive symptoms. The results were reported as odds ratios (OR) and confidence intervals (CI). Subgroup analysis was performed, stratified by gender, smoking, and socioeconomic status. The variance inflation factor (VIF) was used to test multicollinearity, and serious multicollinearity (VIF > 10) was not observed in this study [31]. Differences were considered to be statistically significant if *p*-values were < 0.05. All statistical analysis was performed using SAS software (version 9.4, SAS Institute: Cary, NC, USA).

## 3. Results

We analyzed each of the variables after dividing the cohort according to gender. Table 1 shows the general characteristics of the study population. Among the 58,182 study participants, 5992 male students (20.3%) and 9589 female students (33.4%) had depressed moods. According to the SHSE location categories, the proportion of students with depressed moods was lowest in the “none” group and highest in the “both” group in both boys and girls.

Table 2 gives the adjusted OR (aOR) of depressive symptoms by gender. Regardless of SHSE location and gender, SHSE had a greater association with depression than non-exposure. Furthermore, the association of SHSE and depressive symptoms was highest in the “both” group, followed by the “school” and “home” groups.

Table 3 presents the results of the subgroup analysis according to depressive symptoms. As can be seen from the table, the results differed by location and gender. Stratifying by smoking status, current male smokers and female ex-smokers were the groups in which SHSE both at home and school was most strongly associated with depressive symptoms. In both male and female non-smokers, SHSE at school was most strongly associated with depressive symptoms. In male ex-smokers and current female smokers, SHSE at home was most strongly associated with depressive symptoms. The results of other subgroup analyses were similar to the main results. Notably, according to perceived economic status, there was a stronger association between SHSE location and depressive symptoms in those with high economic status than in those with a low economic status.

**Figure 1:**
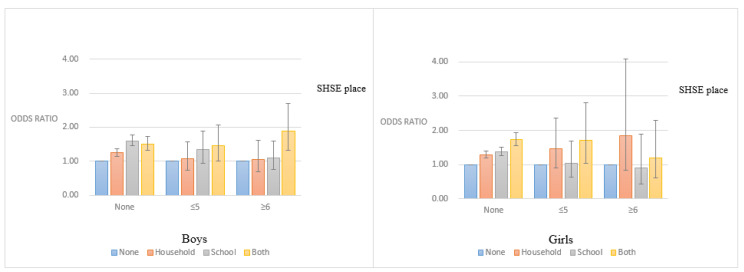
Adjusted odds ratios for depressive symptoms according to SHSE location, classified by the number of cigarettes smoked per day (SHSE: secondhand smoke exposure).

Figure 1 shows the aOR for depressive symptoms according to SHSE location, adjusted for the amount smoked per day. Among non-smokers, the highest aOR for depressive symptoms was in the “school” group for male students and in the “both” group for female students. Furthermore, the aOR for depressive symptoms was lowest in the “none” group and highest in the “both” group among male smokers. Notably, in the “both” group, male students who smoked more than 6 cigarettes/day had a higher aOR for depressive symptoms than those who smoked fewer than 5 cigarettes/day. In contrast to our other results, among female smokers, the “home” group had a higher aOR than the “school” group.

**Figure 2:**
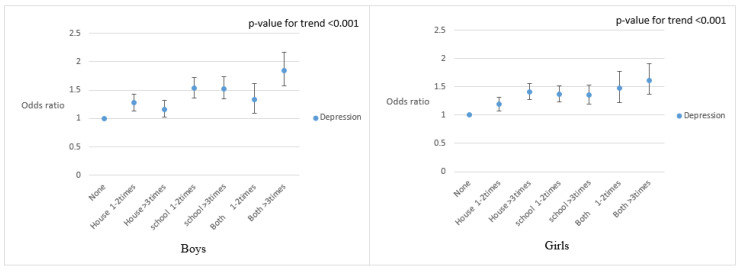
Adjusted odds ratios for depressive symptoms according to SHSE location and the frequency of secondhand smoke exposure. Adolescents who did not experience SHSE were used as reference (SHSE: secondhand smoke exposure).

Figure 2 presents the aOR for depressive symptoms according to SHSE location, adjusted for the amount of SHSE per week. The amount of SHSE and the number of SHSE locations was positively correlated with depressed mood in female students. There was a similar trend seen among male students, with the exceptions of the “1–2 times/week” subgroup in the “both” group and the “over 3 times/week” subgroup in the “home” group.

## 4. Discussion

To date, a number of studies have demonstrated the association between SHSE and depression, for which several mechanisms have been proposed. For example, SHSE could be related to lower dopamine levels [32] or stressful environments [33], which may lead to the development of depression.

In this study, we aimed to determine the association between the location of SHSE and depressive symptoms among adolescents. The results of this study showed similar trends between both genders; the association between SHSE and depressive symptoms was the highest in the “both” group, followed by the “school” and “home” groups. Additionally, in students who were exposed to SHS both at home and at school, there seemed to be an additive effect on the male students and a synergistic effect on the female students.

In contrast, according to the students’ smoking status, the association between SHSE location and depression differed between male and female students. Among non-smokers, the “school” group had the highest OR for depression in male students and the “both” group had the highest OR for depression in female students. This may indicate that male students are more stressed by SHSE at school than at home, which could be connected to their depressive symptoms [34]. In addition, male students could be more easily influenced by their friends than female students [35]. Therefore, male non-smokers may succumb more easily to peer pressure to smoke [36].

Furthermore, the association between SHSE and depression was significant for both genders in all the non-smoking subgroups. These findings confirm those of previous studies, showing that SHSE is associated with depression in non-smoking adolescents [16,17,37]. However, the results differed among current and previous smokers. A possible explanation for this may be due to their life experience. The perception of SHS in current and previous smokers may be different from that of non-smokers due to their nicotine addiction [9].

The present study also focused on adolescents’ socioeconomic status, as demonstrated in the subgroup analysis. First, students who were living with family experienced more depression than those living away from home. This could be because the students who were living away from home were less affected by their family and household status due to the physical distance [38]. Notably, the effects of SHSE were the highest among female students who were living away from home. This result differed from those of other living conditions among female students.

Second, there was a significant association between SHSE location and depression in the medium and high household income groups, but not in the low household income group. This could also be explained by differences in life experience because low household income was associated with depression regardless of SHSE. In addition, adolescents in the low household income group may live in environments in which SHSE is unavoidable [17,39]. Consequently, SHSE may be a familiar part of life and, therefore, not associated with depression. Furthermore, economic status may play more of a role in depression than SHSE itself [40].

This study included smoking students on the premise that adolescents are easily affected by their environment [41]. The results show that male smokers had depressed moods when experiencing SHSE at school and at home, regardless of the amount they smoked. This result supports those of a previous study showed that even if adolescents are smoking, adolescents with SHSE were more depressed than those without SHSE [28]. Furthermore, there was a significant association between SHSE both at school and at home and depressive symptoms among female students who smoked fewer than five cigarettes per day. The results were not significant due to the small number of current smokers, but HSHSE seemed to be more strongly associated with depressive symptoms than SSHSE. Additionally, we found that the frequency of SHSE is positively correlated with depressive symptoms. This finding was also reported by previous studies [28,42].

The main contribution of this study is the confirmation that SHSE location is significantly associated with depressive symptoms, including in former and current smokers. This approach adds to the growing body of knowledge on this subject. In addition, this study demonstrates that adolescents who smoke can also be depressed by SHSE at school or at home. However, the locations associated with depression differ depending on the amount smoked and gender. In addition, since this study was conducted using the extensive population-based survey, our results may be considered as representative of Korean adolescents.

There are several limitations to this study. First, we used cross-sectional data. Therefore, cause and effect and the direction of the relationships observed cannot be determined. Second, we assessed smoking status based on self-reported data, which has proven to be a good indicator of overall tobacco use but may underestimate the use of tobacco in adolescents. Third, we could not assess the extent and duration of SHSE, which would be more effective variables than the frequency of exposure [30]. Fourth, a simple question for detecting depression used in this study was proved feasible in adults but not adolescents [29]. Last, because of a lack of information, we did not consider the family presence of depressive symptoms. Thus, a more comprehensive checklist should be adopted when assessing depressive symptoms in adolescents.

## 5. Conclusions

This study has shown that exposure to SHS in multiple locations is associated with increased depressive symptoms among Korean adolescents regardless of their smoking status. Prior research indicated that exposure to smoke is negatively implicated in adolescent health status [9,10,11]. Likewise, adolescents are greatly affected by their surrounding environments and depression in adolescence increases the risk of later adverse psychosocial outcomes, such as later major depression and anxiety disorders [42]. Therefore, healthier environments are necessary for adolescents. Stricter smoking regulations and policies enforcing smoking bans in the household and schools should be implemented. In addition, according to previous research, developing national smoking prevention programs including peer-to-peer intervention is needed [43]. Similarly, it is necessary to provide local-level access to smoking-related educational programs for families of adolescents.

## Figures and Tables

**Table 1:** General characteristics of the study population.

Variables	Depressive Symptoms													
	Boys							Girls						
	Total		Yes		No		*p-*Value	Total		Yes		No		*p-*Value
	*N*	%	*N*	%	*N*	%		*N*	%	*N*	%	*N*	%	
Total (*n* = 58,182)	29,481	100.0	5992	20.3	23,489	79.7		28,701	100.0	9589	33.4	19,112	66.6	
SHSE locations ^a^							<0.0001							<0.0001
Both	2252	7.6	711	31.6	1541	68.4		2024	7.1	1003	49.6	1021	50.4	
School	3467	11.8	972	28.0	2495	72.0		3341	11.6	1326	39.7	2015	60.3	
Home	4384	14.9	1003	22.9	3381	77.1		4991	17.4	1941	38.9	3050	61.1	
None	19,378	65.7	3306	17.1	16,072	82.9		18,345	63.9	5319	29.0	13,026	71.0	
Age (years)							<0.0001							<0.0001
12	2296	7.8	380	16.6	1916	83.4		2351	8.2	645	27.4	1706	72.6	
13	4901	16.6	812	16.6	4089	83.4		4706	16.4	1458	31.0	3248	69.0	
14	5070	17.2	978	19.3	4092	80.7		4821	16.8	1648	34.2	3173	65.8	
15	4788	16.2	1005	21.0	3783	79.0		4736	16.5	1554	32.8	3182	67.2	
16	4928	16.7	1046	21.2	3882	78.8		4555	15.9	1572	34.5	2983	65.5	
17	5117	17.4	1207	23.6	3910	76.4		5222	18.2	1891	36.2	3331	63.8	
18	2381	8.1	564	23.7	1817	76.3		2310	8.0	821	35.5	1489	64.5	
Grade							<0.0001							<0.0001
Middle school 1st–3rd	14,837	50.3	2737	18.4	12,100	81.6		14,407	50.2	4619	32.1	9788	67.9	
High school 1st–3rd	14,644	49.7	3255	22.2	11,389	77.8		14,294	49.8	4970	34.8	9324	65.2	
Living status							<0.0001							0.0011
Living with family	27,800	94.3	5563	20.0	22,237	80.0		27,226	94.9	9038	33.2	18,188	66.8	
Living away from family	1681	5.7	429	25.5	1252	74.5		1475	5.1	551	37.4	924	62.6	
Perceived economic status							<0.0001							<0.0001
Low	3703	12.6	1062	28.7	2641	71.3		3969	13.8	1776	44.7	2193	55.3	
Middle	13,013	44.1	2492	19.2	10,521	80.8		14,010	48.8	4472	31.9	9538	68.1	
High	12,765	43.3	2438	19.1	10,327	80.9		10,722	37.4	3341	31.2	7381	68.8	
Smoking status							<0.0001							<0.0001
Never	23,611	80.1	4253	18.0	19,358	82.0		26,430	92.1	8416	31.8	18,014	68.2	
Ex-smoker	3396	11.5	881	25.9	2515	74.1		1268	4.4	607	47.9	661	52.1	
Current smoker	2474	8.4	858	34.7	1616	65.3		1003	3.5	566	56.4	437	43.6	
Family smoking status							<0.0001							<0.0001
None	13,562	46.0	2549	18.8	11,013	81.2		12,562	43.8	3833	30.5	8729	69.5	
Parents	13,176	44.7	2807	21.3	10,369	78.7		13,083	45.6	4688	35.8	8395	64.2	
Others	2743	9.3	636	23.2	2107	76.8		3056	10.6	1068	34.9	1988	65.1	
Friends’ smoking status							<0.0001							<0.0001
None	15,422	52.3	2411	15.6	13,011	84.4		20,420	71.1	5895	28.9	14,525	71.1	
Some	11,180	37.9	2630	23.5	8550	76.5		7091	24.7	3053	43.1	4038	56.9	
Most/All	2879	9.8	951	33.0	1928	67.0		1190	4.1	641	53.9	549	46.1	
Experience drinking alcohol							<0.0001							<0.0001
Yes	13,207	44.8	3476	26.3	9731	73.7		10,678	37.2	4543	42.5	6135	57.5	
No	16,274	55.2	2516	15.5	13,758	84.5		18,023	62.8	5046	28.0	12,977	72.0	
Physical activity/week (hours)							<0.0001							<0.0001
0	8050	27.3	1408	17.5	6642	82.5		12,836	44.7	4072	31.7	8764	68.3	
1–4	15,275	51.8	3222	21.1	12,053	78.9		13,725	47.8	4693	34.2	9032	65.8	
5–7	6156	20.9	1362	22.1	4794	77.9		2140	7.5	824	38.5	1,316	61.5	
Academic score							<0.0001							<0.0001
Low	9189	31.2	2239	24.4	6950	75.6		9238	32.2	3659	39.6	5579	60.4	
Middle	8332	28.3	1605	19.3	6727	80.7		8706	30.3	2736	31.4	5970	68.6	
High	11,960	40.6	2148	18.0	9812	82.0		10,757	37.5	3194	29.7	7563	70.3	
Stress status							<0.0001							<0.0001
High	9305	31.6	3764	40.5	5541	59.5		14,166	49.4	7188	50.7	6978	49.3	
Low	20,176	68.4	2228	11.0	17,948	89.0		14,535	50.6	2401	16.5	12,134	83.5	

^a^ The places where adolescents experienced secondhand smoke exposure.

**Table 2:** Factors associated with depressive symptoms.

Variables	Depressive Symptoms			
	Boys		Girls	
	aOR	95% CI	aOR	95% CI
SHSE locations ^a^				
Both	1.61 *	(1.44–1.80)	1.72 *	(1.54–1.91)
School	1.53 *	(1.39–1.67)	1.36 *	(1.25–1.48)
Home	1.23 *	(1.12–1.35)	1.30 *	(1.20–1.40)
None	1.00		1.00	
Age (years)				
12	1.00	(0.83–1.10)	1.00	
13	0.96		1.05	(0.93–1.18)
14	1.01	(0.87–1.16)	1.08	(0.96–1.21)
15	1.08	(0.92–1.26)	1.04	(0.91–1.19)
16	1.13	(0.92–1.38)	1.17	(0.98–1.39)
17	1.17	(0.96–1.44)	1.20 *	(1.01–1.43)
18	1.12	(0.90–1.39)	1.13	(0.93–1.36)
Grade				
Middle school 1st–3rd	1.34 *	(1.16–1.56)	1.37 *	(1.20–1.56)
High school 1st–3rd	1.00		1.00	
Living status				
Living with family	1.00		1.00	
Living away from family	0.84	(0.74–0.95)	0.91	(0.80–1.02)
Perceived economic status				
Low	1.21 *	(1.10–1.33)	1.22 *	(1.12–1.33)
Middle	0.94	(0.88–1.00)	0.95	(0.90–1.01)
High	1.00		1.00	
Smoking status				
Never	1.00		1.00	
Ex-smoker	1.14 *	(1.03–1.25)	1.23 *	(1.08–1.40)
Current smoker	1.33 *	(1.19–1.50)	1.26 *	(1.08–1.48)
Family smoking status				
None	1.00		1.00	
Parents	0.92	(0.86–0.99)	0.95	(0.89–1.01)
Others	1.09	(0.98–1.21)	1.03	(0.94–1.13)
Friends’ smoking status				
None	1.00		1.00	
Some	1.36 *	(1.26–1.46)	1.40 *	(1.31–1.49)
Most/All	1.55 *	(1.37–1.75)	1.60 *	(1.38–1.85)
Experience drinking alcohol				
Yes	1.44 *	(1.34–1.54)	1.43 *	(1.35–1.52)
No	1.00		1.00	
Physical activity/week (hours)				
0	0.75	(0.69–0.82)	0.78	(0.71–0.87)
1–4	0.94	(0.87–1.02)	0.91	(0.82–1.01)
5–7	1.00		1.00	
Academic score				
Low	1.21 *	(1.12–1.30)	1.28 *	(1.20–1.37)
Middle	1.08 *	(1.00–1.16)	1.09 *	(1.02–1.16)
High	1.00		1.00	
Stress status				
High	5.14 *	(4.84–5.47)	4.82 *	(4.56–5.10)
Low	1.00		1.00	

aOR: adjusted ORs; CI: confidence intervals; *: statistically significant. ^a^ The places where adolescents experienced secondhand smoke exposure.

**Table 3:** Subgroup analysis according to depressive symptoms.

Variables	Depressive Symptoms						
	SHSE Locations ^a^						
	None	Both		School		Home	
	aOR	aOR	95% CI	aOR	95% CI	aOR	95% CI
Boys							
Smoking status							
Never	1.00	1.56 *	(1.35–1.80)	1.63 *	(1.47–1.82)	1.23 *	(1.11–1.37)
Ex-smoker	1.00	1.32	(0.99–1.77)	1.43 *	(1.12–1.82)	1.30 *	(1.02–1.64)
Current smoker	1.00	1.73 *	(1.35–2.22)	1.20	(0.94–1.54)	1.09	(0.82–1.43)
Living status							
Living with family	1.00	1.61 *	(1.43–1.81)	1.55 *	(1.41–1.70)	1.23 *	(1.12–1.36)
Living away from family	1.00	1.45	(0.98–2.14)	1.24	(0.85–1.81)	1.21	(0.83–1.78)
Perceived economic status							
Low	1.00	1.49 *	(1.15–1.93)	1.47 *	(1.18–1.84)	1.06	(0.85–1.32)
Middle	1.00	1.66 *	(1.40–1.97)	1.56 *	(1.35–1.80)	1.31 *	(1.14–1.50)
High	1.00	1.61 *	(1.35–1.93)	1.51 *	(1.31–1.74)	1.24 *	(1.07–1.43)
Girls							
Smoking status							
Never	1.00	1.73 *	(1.54–1.94)	1.38 *	(1.26–1.51)	1.28 *	(1.18–1.39)
Ex-smoker	1.00	1.78 *	(1.18–2.69)	1.32	(0.91–1.93)	1.33	(0.96–1.84)
Current smoker	1.00	1.47 *	(1.00–2.17)	1.00	(0.67–1.48)	1.53 *	(1.03–2.28)
Living status							
Living with family	1.00	1.77 *	(1.59–1.98)	1.36 *	(1.25–1.48)	1.31 *	(1.21–1.42)
Living away from family	1.00	1.04	(0.68–1.59)	1.29	(0.91–1.83)	1.06	(0.73–1.54)
Perceived economic status							
Low	1.00	1.64 *	(1.29–2.08)	1.49 *	(1.21–1.83)	1.13	(0.94–1.35)
Middle	1.00	1.68 *	(1.44–1.97)	1.33 *	(1.17–1.50)	1.27 *	(1.14–1.42)
High	1.00	1.81 *	(1.50–2.19)	1.34 *	(1.17–1.54)	1.47 *	(1.28–1.69)

aOR: adjusted ORs; CI: confidence intervals; *: statistically significant. ^a^ The locations where adolescents experienced secondhand smoke exposure.
